# Association of serum brain derived neurotropic factor with duration of drug-naive period and positive-negative symptom scores in drug naive schizophrenia

**DOI:** 10.1371/journal.pone.0189373

**Published:** 2017-12-29

**Authors:** Abdurrahim Bakirhan, Safak Yalcin Sahiner, Ismail Volkan Sahiner, Yasir Safak, Erol Goka

**Affiliations:** 1 Department of Psychiatry, Elbistan State Hospital, Kahramanmaras, Turkey; 2 Department of Psychiatry, Numune Research and Training Hospital, Ankara, Turkey; 3 Department of Psychiatry, Diskapi Yildirim Beyazit Research and Training Hospital, Ankara, Turkey; Chiba Daigaku, JAPAN

## Abstract

**Introduction:**

The aim of this study was to compare the serum brain derived neurotropic factor (BNDF) levels of patients with schizophrenia who had never received an antipsychotic treatment with those of a control group. Also, to analyze the relationship between the Positive and Negative Symptom Scale (PANSS) scores and BDNF levels of the patients during the period they were drug-naive.

**Materials and methods:**

The sample of the study comprised patients who presentedto the Psychiatry Clinic and were admitted after a distinctive schizophrenia diagnosis was made in accordance with the fourth edition of the Diagnostic and Statistical Manual of Mental Disorders (DSM-IV-TR) diagnosis classification and who were not using and never had any antipsychotic medicine. A total of 160 participants were included in the study, 80 of whom had schizophrenia patients and 80 constituted the age- and sex-matched healthy control group. Before the start of the treatment, the serum samples to be checked for the BDNF levels were collected from the patients.

**Results:**

The difference between the average BDNF levels of the groups were statistically significant (t = -5.25; p˂.001). An analysis as to whether there was a relation between the BDNF levels and the drug-naïve duration indicated no correlations. An examination of the relationship between PANSS scores and BDNF levels of the patients yielded no correlations.

**Discussion:**

Serum BDNF levels seem to be one of the indicators of schizophrenia and its progress; nevertheless, we still do not have sufficient information about this neurotropic factor. In light of our study, the neurodevelopmental changes that occur at disease onset of the illness prominently affect the progress of the illness, which highlights the importance of the treatment in the early stages.

## Introduction

Schizophrenia has a heterogeneous nature as a disease yet its etiopathogenesis has yet to be thoroughly explained. It causes significant loss of faculties and chronic advance of the disease brings a heavy financial and psychological burden on the individual, family, and society [1].To date, many hypotheses have been asserted in order to cast light on the etiology of schizophrenia [2]. One of these, on which most emphasis has been placed, is the neurodevelopmental hypothesis. The neurodevelopmental hypothesis claims that any disorders that occur in neuronal migration, neuronal connections and neural plasticity cause structural abnormalities in specific areas of the brain which play a significant role in the development of schizophrenia [3]. One of the important factors behind such disorders, brain derived neurotropic factor (BDNF), causes neurons to be produced, reproduce, and survive [4–6].There is a sizeable amount of evidence defining the probable relationship between the pathophysiology of schizophrenia and BDNF, which is actually related to the conservation of neurons [7–8].

Even though there is much evidence in different resources proving that levels of BDNF vary in the brains of patients with schizophrenia, the results are debatable. Many studies reported that blood BDNF levels of patients with schizophrenia varied. For example, when compared with healthy volunteers, a significant decrease was found in the serum BDNF levels of patients with schizophrenia [9–12].In contrast, other studies report increasing levels of BDNF in patients with schizophrenia [13–14]. Apart from these studies, there are studies in the literature where no differences were observed in the serum BDNF concentrations of controls and patients [15–17]. Decreased BDNF levels in patients with schizophrenia were recently confirmed in a metaanalysis that was comprised 41 studies and more than 7000 participants. It was determined that this decrease was significant at the first episode and become apparent during the progression of the illness [18].

Though there are limited amount of data derived from drug-naïve patients with schizophrenia in literature, it was detected that drug-naïve patients had lower BDNF levels compared with those of controls [19–20]. In the same metaanalysis mentioned above, regardless of psychiatric medication in schizophrenia, lower BDNF levels were reported and no relationship was confirmed between the levels of BDNF and the amount of medicine used [18]. In addition, low BDNF levels were detected in patient with first episode schizophrenia in many studies[3],[11–12],[21–24].Different results were also found also in the few studies that investigated the relationship between the serum BDNF levels of the patients and the duration of drug naivety. A negative correlation [24] or a significant correlation [3]was detected between the duration of drug naivety and the serum BDNF levels.

Quite different results were gathered in studies that inspected the relationship between BDNF levels and the intensity of the positive and negative symptoms or general psychopathology (Positive and Negative Symptom Scale-PANSS). Although a positive correlation between PANSS positive subscale scores and serum BDNF levels has been detected in some studies, in other studies where a significant relation was found in PANSS negative subscale scores and serum BDNF levels [3], a negative correlation was found between both positive and negative subscale scores and serum BDNF levels [12]. On the other hand, no significant relation was detected in some studies between serum BDNF levels and PANSS subscale scores [16]. A recent metaanalysis demonstrated that decreased peripherical BDNF levels were not related with severity of positive and negative symptoms [18].

In view of the given literature, our goal in this study was to compare the serum BDNF levels of patients with schizophrenia who had never received an antipsychotic treatment with those of a control group and to analyze the relationship between the PANSS scores and BDNF levels of the patients during the period they were drug-naïve.

## Materials and methods

### Sample

The sample group of the study comprised patients who presented to Ankara Numune Research and Training Hospital Psychiatry Clinic as outpatients between 2012 and 2014and were admitted with a distinctive schizophrenia diagnosis in accordance with the fourth edition of the Diagnostic and Statistical Manual of Mental Disorders (DSM-IV-TR) diagnostic criteria, and who was not using and never had used any antipsychotic medicine. A total of 160 participants were included in the study.80 of whom had schizophrenia patients and 80 were the healthy age- and sex-matched controls. The patients, the patient’s relatives and healthy volunteers were informed about the study and their written consent was obtain.

The inclusion criteria for patients in the study were first-time diagnosis of schizophrenia (according to DSM diagnostic criteria), and informed consent given by the patient or a close relative. The exclusion criteria for the study were current or previous use of antipsychotic drugs, presence of metabolic or endocrinologic illness, mental retardation, with another axis I diagnosis, alcohol or substance abuse/addiction, and patients who took other medication for other reasons (e.g., antidepressants, mood stabilizers, thyroid hormone, corticosteroids).

The control group was formed by volunteers who matched the patients with schizophrenia patients in terms of age and sex who were mentally and physically healthy and had no psychotic disorder history in their first-degree relatives.

### Data collection and laboratory tools

*Socio-demographical information form (S1 and S2 Files)*: This is a semi-structured form used to capture the socio-demographic features of the patients and control group that participated in the study. Using this form, questions about the age, sex, marital status, education level, job, income, duration of the illness, family history of the disease, substance or drug use, smoking of the subject were asked.

*Semi-structured clinical interview form for Axis-1 diagnosis*: *structured clinical interview for DSM-4-TR (SCID-I)*: This is a clinical interview form developed by First et al [25] for DSM-4 axis 1 disorders. Through this form the diagnostic evaluation is implemented in a standardized manner, thereby enhancing the validity of the diagnosis [25].

*Positive and Negative Symptom Scale (PANSS)*: Developed by Kay et al [26], this scale is used to measure the level, distribution and intensity variation of positive and negative symptoms of schizophrenia in a subject. It comprises 3 subscales and 30 items. These subscales are positive symptoms, negative symptoms and general psychopathy.

*Measuring BDNF levels*: Serum BDNF levels were measured using commercial enzyme-linked immunosorbent assay (ELISA) kits (BOSTER Immunoleader, Boster Biological Technology Co., Ltd., CA, USA. Code: EK0307, Lot No: 361082901). Within the studies is CV%; 3.5–4.9 and between the studies is CV%;7.2–7.9. The sensitivity is <2pg/mL and the measuring interval is 31,2-2000pg/mL. The test was performed appropriately in accordance with the principals of the kit. Some kits measure mature BDNF, whereas other measure Pro-BDNF levels. The kits that we used measured mature BDNF levels. The financial resource of the kits used in the study was Scientific Research Support Fund of Ankara Numune Research and Training Hospital.

## Method

Patients who presented to Ankara Numune Training and Research Hospital Psychiatry Clinic for treatment and were not currently using and had ever used any antipsychotic medication were directed to the study physician. The period that the patients were not drug naïve starting from the beginning of the symptoms of the illness were detected by taking detailed anamnesis from the relatives of the patient. The patients and the relatives were informed of the study and 2signed consent forms were collected, one from the patient and one from the close relative. The study was reviewed and approved by Ankara Numune Research and Training Hospital Ethics Committee for researchers who meet the criteria for access to confidential data.

In the psychiatric interview conducted with the patients in accordance with the Structured Clinical Interview for DSM-IV Axis I Disorders (SCID-I) and DSM-IV, the compliance of the diagnosis with “schizophrenia” in axis I, and the history of symptoms for six months or more were verified. The physical examination of the phenomena for the inclusion in the study criteria was conducted. The information form of socio-demographic questions was completed by the participants. The psychiatric interview were performed by the researcher and clinical scales were evaluated by the physicians. PANSS scale was applied to evaluate the clinical condition of the patients. Serum samples to be checked for BDNF levels were collected from the patients before the start of the treatment.

The venous blood samples of the subjects and control group were collected in biochemistry tubes in the morning between 08.00 AM and 09.00 AM under the same conditions and rested for 30 minutes for the bloods to coagulate. The bloods were then centrifuged for 10 minutes at3500 rpm in room temperature. The serum part of the blood was separated and stored in 70°C until the time of the measurement. After this process, the differences in serum BDNF in the patient groups were determined

### Data analysis

All the collected data were numerically coded and assessed using the Statistical Program for Social Sciences for Windows (SPSS) version 15.0. For the definitive statistics, frequency distribution was calculated and for continuous variables the arithmetic average and standard deviation value were calculated. Student’s t-test was used for the comparison of parametric data in binary groups, and the Mann-Whitney U test was used for non-parametric data. The Kruskal-Wallis test was used for the comparison of the values of more than 2 groups. Tukey’s multiple comparison test was used for the groups that were significant, and the differences between the groups were tested in pairs. Additionally, in order to perceive the relation between the variables, Pearson’s correlation test was used. The significance level was established as .05 and the *p* value was compared with the significance value as calculated using SPSS. The power of our study was calculated with post-hoc power calculator of G-Power for Mac OS X (version 3.1.9.2; Universitat Düsseldorf, Germany). According to BDNF results of previously and our studies, the power of our study was detected as 99% with alpha value as .05 for our sample size (n: 80 each branch).

## Results

When the demographic features of the participants were analyzed; 33 (41.2%) of both the patient and the control group were women, and 47 (58.8%) were men. The mean age of the participants was30.59±9.15 years (range, 17–59 years).Both groups were similar in terms of age and sex. Sociodemographic features of both groups are presented in detail in Table 1.

**Table 1:** Demographical features of the participating patients and control group.

Demographical Features		Patient Group N = 80	Control Group N = 80	p
**Sex**	**Men** **Women**	47 (%58.8) 33 (%41.2)	47 (%58.8) 33 (%41.2)	
**Age (average)** **(year)**		31.08±9.37 (17–59)	29.65±8.78 (17–59)	.63

Student t test, p < .05

When the clinical features of the participating patients were analyzed; the average age of illness occurrence was 26.77±7.28 years and the average duration of the illness was 4.25±5.29 years. The clinical feature and scores of both groups are shown in detail in Table 2.

**Table 2:** The clinical features of the patients participating in the study.

Clinical Features	Patient group N = 80 Mean±SD
Age atonset of illness (year)	26.77±7.28
Average duration of illness (year) (the drug-naïve duration)	4.25±5.29
PANSS Negative	30.42±4.10
PANSS Positive	28.92±3.37
PANSS General psychopathology	51.25±4.29
PANSS Total	110.63±9.76

**Fig 1:**
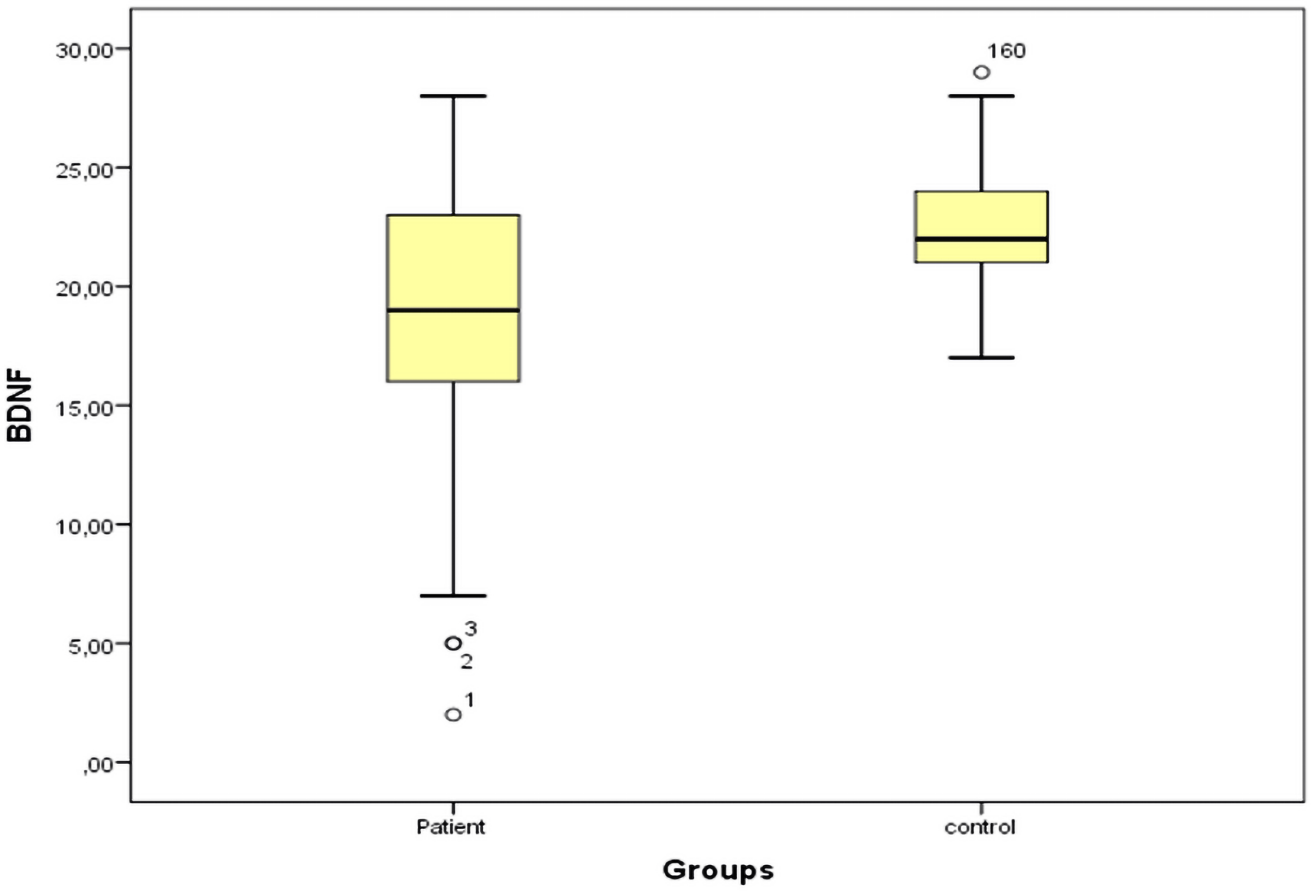
Dot plot of control and patients groups.

When the BDNF levels of the both patients and controls were analyzed, the average BDNF levels were 18.75±5.62ng/mL, and 22.35±2.44ng/mL, respectively (Fig 1). When average BDNF levels of both groups were compared, the difference between the average the BDNF levels of the groups were statistically significant (t = -5.25; p˂.001). An analysis as to whether there was a relation between the BDNF levels and the drug-naïve duration indicated no correlations (r = 0.117; p = .302).

An examination of the relationship between PANSS scores and BDNF levels of the patients yielded no correlations (Table 3).

**Table 3:** The relation between BDNF levels and PANSS scores.

	BDNF
**PANSS Negative**	r = -.003 p = .979
**PANSS Positive**	r = -.157 p = .164
**PANSS General Psychopathology**	r = -.009 p = .938
**PANSS Total**	r = -.058 p = .610

Pearson Correlation

With a categorization of the durations during which the patients were drug-naïve, as 0–1 year, 1–5 years and more than 5 years, the analysis of the BDNF levels of the 3 groups indicated no differences (p = .305). In terms of PANSS scores between the groups the statistical differences were detected in negative, general psychopathology, and total scores (Table 4).

**Table 4:** Comparison of BDNF and PANSS scores between the different drug-naïve duration groups.

	0–1 year (n = 30) Mean±SD	1–5 years (n = 30) Mean±SD	More than 5 years (n = 20) Mean±SD	p
**BDNF (ng/ml)**	17.30±6.22	20.20±4.42	18.75±5.97	.305
**PANSS Negative**	28.43±3.61	31.63±2.98	31.60±5.15	.003*
**PANSS Positive**	28.70±3.59	29.46±3.20	28.45±3.36	.537
**PANSS General Psychopathology**	49.33±4.07	52.13±3.51	52.80±4.80	.004*
**PANSS Total**	106.46±9.17	113.23±7.87	113.00±11.38	.013*

Kruskal Wallis Test

*p˂.05

The comparison between the group that was drug-naïve for a year and the group that was drug-naïve for more than 5 years showed no significant differences in terms of BDNF levels (z = -0.843; p = .399). In terms of BDNF levels and PANSS scores, no significant difference was detected in the comparison between the group that was drug-naïve for 1–5 years and the group that was drug-naïve for more than 5 years (z = -0.625; p = .532). In the analysis to detect relations between BDNF levels and the time patients with the illness for less than a year spent drug-naïve, no correlation was found (r = 0.143; p = .452).

## Discussion

The neurodevelopmental model of schizophrenia is one of the most debated subjects of our era. BDNF is one of the important neurotropic factors that plays a role in the neurodevelopmental model and schizophrenia pathophysiology [27].Although the source of BDNF in the serum has not yet been clearly discovered because blood can pass through the brain barrier in both directions [28], the serum BDNF gives us have an idea about cortical BDNF [29]. Serum BDNF levels seem to be one of the indicators of schizophrenia and its progress; nevertheless, there is insufficient information about this neurotropic factor.

The serum BNDF levels of the schizophrenia group were significantly lower than that of the control group in our study, similar to studies in the literature [3],[11],[20],[22–23],[30]. In the study by Buckley et al [11], the serum BDNF levels of 15 patients with schizophrenia who were drug-naïve and 14 healthy controls were compared and the serum BDNF levels of the patients with schizophrenia were lower. In a more extensive study conducted by Chen et al [3], serum BDNF levels of 88 patients with schizophrenia who had received no antipsychotic treatments and a control group (n = 90) were compared in an effort to discover the relation between BDNF and schizophrenia subtypes; serum BDNF levels of patients with schizophrenia were significantly lower than those of the control group. In another recent study by Marianthi Sotiropoulou et al [20], serum BDNF levels of 50 patients with schizophrenia who were treatment naïve were compared with a control group of 50 healthy people; the serum BDNF levels of the group with schizophrenia were detected to be statistically significantly lower than in the control group.

There are also some studies in the literature whose results conflict with ours [13–15]. Shimizu et al [15] investigated 40 patients with schizophrenia, 15 of which had never been on antipsychotic treatment and 25 still under treatment, and a control group of 40 healthy people. The serum BDNF levels of these 3 groups were compared and no significant difference was found between the groups. The difference in the results of this study might have been caused by the significantly smaller number of patients who had never received antipsychotic treatment compared with our study. In a study by Reis et al [14], serum BDNF levels of 40 male patients with schizophrenia who were receiving treatment were compared with a control group of 20 healthy people. The results showed the serum BDNF levels of the group with schizophrenia were significantly higher than the control group [14]. Another study compared serum BDNF levels of 60patients with schizophrenia who were undergoing treatment with 26 healthy people and 30 euthymic patients who were bipolar [13]. The results indicated that the serum BDNF levels of patients with schizophrenia were significantly higher than that of both the bipolar group and the control group [13]. The reason why our results are different than the two other studies might be related to the patients’ use of antipsychotic treatments in both cases, the intensity of the illness, the patient profile and treatment, and the genetic background of the patients because both studies were conducted in Brazil.

Even though the average illness occurrence age of the patients in our study is compatible with the literature, the drug-naïve duration (51±61.39 months) was significantly longer in our study that those in the literature. This may be due to the fact that in our country the symptoms of schizophrenia are received by society more normally than in other countries and the patients are treated later. When we analyzed as to whether there was a relation between serum BDNF levels and the drug-naïve duration, no statistically significant correlation was detected. It is possible to read the lack of correlation as a decrease of serum BDNF levels that occurred along with the on set of the illness but did not continue during the whole duration of the illness. Contrato our results, Rizos et al [24] found a negative correlation between the drug-naïve duration of the patients with schizophrenia and the serum BDNF levels.

When the treatment duration of the patients in our study was taken as 0–1 year and 1–5 years, it was observed that serum BDNF levels of the drug-naïve patients for 0–1 year were lower (17.30±6.22ng/mL). The serum BDNF levels of the group that was drug-naïve for 1–5 years were (20.20±4.42 ng/mL). These results were statistically significant. In the study by Rizos et al [24], the drug-naïve duration was an the average 6.4±5.26 months, which was significantly lower than the drug-naïve duration in our study (51±61.39 months). Additionally there is a clear difference between the patient numbers in our study (n = 80) compared with the study by Rizos et al [24] (n = 37). These findings clearly show that the serum BDNF levels significantly decrease at the onset of the illness but do not proceed to decline in the following. Contrary to Rizos et al [24], in our study, in the group in which patients were drug-naïve for less than a year (n = 30), no significant difference was detected between the serum BDNF levels. In a similar study by Chen et al [3] no significant relation was determined between the serum BDNF levels of the patients and the drug-naïve duration, as was revealed in our study.The drug-naive duration (23.4±19.1 months)and number of patients (n = 88) in their study was closer to our study than in Rizos et al’s [24] and significantly longer that the other studies on the subject [3].The reason why the results are different as such may be because of a different BDNF genetic polymorphism in Eastern and Western societies.

The analysis on the relation between PANSS scores and serum BDNF levels of the patients revealed no correlations. The results in the related researches in the literature are varied. A relation between the PANSS score and serum BDNF levels was investigated in the study by Chen et al [3] and although a positive correlation was discovered between PANSS positive subscale scores and serum BDNF levels, no significant relation was not found between PANSS negative subscale scores and serum BDNF levels. Rizos et al [23] researched the relationship between serum BDNF levels and PANSS subscale scores in their study and detected a negative correlation between serum BDNF levels and both positive and negative subscale scores. Reis et al [14] reported no significant relation between serum BDNF levels and PANSS positive subscales; however, they did detect a positive correlation between PANSS negative subscale scores and serum BDNF levels. In the study by Huang and Lee [16], as in our study, no significant relation was detected between serum BDNF level and PANSS subscale scores. The reason for these differences in the study results is not clearly known. Still, the variation might be related to the difference in the clinical conditions of the patients, duration of the illness or the ethnic variation of allele frequency depending on BDNF gene polymorphism.

To mention the limitations of our study, although the size of the sample is sufficient, a larger sample would have given additional accuracy. The cultural values and religious beliefs of our society make the schizophrenia more acceptable, which delays the presentation of patients to hospitals and extends the drug-naive duration. This extensional so created the opportunity to analyze patients with schizophrenia who have been drug-naïve for a long time. Although serum BDNF levels indirectly indicate cortical BDNF levels, a method that measures cortical BDNF would add to the strengths of the study. Although BDNF levels in human serum can be measured using commercially available human BDNF ELISA kits early versions of these kits were unable to distinguish between proBDNF and mature BDNF [31]. Yoshida et al [32] reported that serum levels of proBDNF and mature BDNF in healthy subjects were measurable using newly available human proBDNF and BDNF ELISA kits.

## Conclusion

In the light of our study, the neurodevelopmental changes that occur at the onset of the illness prominently affect the progress of the illness, which highlights the importance of the treatment in the early stages. Information in the literature about the subject is both limited and contradictory. That the drug-naïve duration of patients with schizophrenia is longer than Western countries facilitated an analysis between drug-naïve duration and serum BDNF levels. The results reveal that although BDNF levels play an important role in schizophrenia, how much and in what ways this role changes during the drug-naïve duration remains a subject in need of more research.

Future studies should be conducted to inspect how the drug-naïve duration affects schizophrenia and how this affects affect the progress of the illness. This would also help us understand the role of factors that affect the etiology of schizophrenia.

## Supporting information

S1 File Socio-demographical information form-English. (PDF)

S2 File Socio-demographical information form-Turkish. (PDF)
