# Supplementary material for: Association of serum brain derived neurotropic factor with duration of drug-naive period and positive-negative symptom scores in drug naive schizophrenia
Source: PLoS One. 2017 Dec 29;12(12):e0189373. doi: 10.1371/journal.pone.0189373 (PMC5747443; doi:10.1371/journal.pone.0189373)
Supplement: S1 File — (PDF) [file pone.0189373.s001.pdf]

S1 File. Socio-demographic information form

Name- Surname	
File Number	
Age	
Gender	1. Female ( )
	2. Male ( )
Telephone Number	
Address	
Date	....../……/…...
1.Martial Status	1.Single( )
	2.Married( )
	3.Divorced( )
	4.Widowed( )
2.Education	1.Illiterate( )
	2.Literate( )
	3.Primary school( )
	4.Secondary school( )
	5.High school( )
	6.Univesity( )
3.Living withwhom	1.Parents( )
	2.Relatives( )
	3.Wife/Husbandandchildren( )
	4.Alone( )
	5.Instutionalized individual( )
	6.Homeless( )
4.Number of brothersandsisters	1.None( )
	2.One( )
	3.Two( )
	4.Three( )
	5.Four andover( )

5.Region of residence	1.City( )
	2.Country( )
	3.Village( )
6.Working status	1.Working( )
	2.Not-working( )
7. Occupation	1.Officer ( )
	2. Employee ( )
	3. Artisan ( )
	4. Retired ( )
	5. Other ( )
8.Monthly income of thepatient’shome (TL)	1. 0-500 TL ( )
	2. 500-1000 TL ( )
	3. 1000-1500 TL ( )
	4. 1500-2000 ( )
	5. 2000-….. ( )
9. Age at onset of illness	
10. Duration of illness	………./mounth
11. Duration of illnesswithouttreatment	………./mounth
12. Thelastperiod of regulartreatment	………./mounth
13. Number of hospitalizations	1.None( )
	2.One( )
	3.Two( )
	4.Three( )
	5.Four andover( )
14. Familyhistory of psychiatricillness	1.No ( )
	2.Yes ( )
15. Familyhistory of psychiatricdisorder	1.No ( )

	2.Yes ( )
16. Familyhistory of mooddisorder	1. No ( )<br>2. Yes ( )
17. History of suicide	1.No ( )<br>2.Yes ( )
18. Self mutilizm	1.No ( )<br>2.Yes ( )
19. History of comorbidillnessordrugabuse	0. Alcohol<br>1. Drugabuse…whichone?<br>2. Smoking<br>3. Pregnancy<br>4. Hepatitis<br>5. HIV<br>6. Other………
20.Is thepatientcurrentlyreceivingtreatment?	1. No ( )<br>2. Yes ( )
21.Current clinicalstatus	1. Exacerbation ( )<br>2. Partialremission ( )<br>3. Full remission ( )
22.PANSS score	1. Positivescale:<br>2. Negativescale:<br>3. General psychopatologyscale:<br>4.Total score:
23.Serum BDNF level:	
