# Supplementary material for: Association of serum brain derived neurotropic factor with duration of drug-naive period and positive-negative symptom scores in drug naive schizophrenia
Source: PLoS One. 2017 Dec 29;12(12):e0189373. doi: 10.1371/journal.pone.0189373 (PMC5747443; doi:10.1371/journal.pone.0189373)
Supplement: S2 File — (PDF) [file pone.0189373.s002.pdf]

S2 File. Sosyodemografik veri formu

Adı Soyadı	
Dosya no	
Yaş	
Cinsiyet	1. Kadın ( )
	2. Erkek ( )
Telefon	
Adres	
Tarih	....../……/……
1.Medeni durum	1.Bekar( )
	2.Evli( )
	3.Boşanmış( )
	4.Dul( )
2.Eğitim Durumu	1.Okur yazar değil( )
	2.Okur yazar( )
	3.İlkokul mezunu( )
	4.Ortaokul mezunu( )
	5.Lise mezunu( )
	6.Üniversite ve üzeri( )
3.Kimlerle yaşadığı	1.Ebeveynleri ile( )
	2.Yakınları ile( )
	3.Eş ve çocukları ile ( )
	4.Yalnız( )
	5.Sosyal kurum( )
	6.Evsiz( )
4.Kardeş sayısı	1.Kardeşi yok( )
	2.Bir kardeşi var( )
	3.İki kardeşi var( )
	4.Üç kardeşi var( )
	5.Dört ve üzeri kardeşi var( )

5.Yaşadığı bölge	1.il( )
	2.ilçe( )
	3.Köy( )
6.Çalışma durumu	1.Çalışıyor( )
	2.Çalışmıyor( )
7. Meslek	1.Memur ( )
	2. İşçi ( )
	3. Esnaf ( )
	4. Emekli ( )
	5. Diğer ( )
8.Yaşadığı hanenin aylık gelir miktarı (TL)	1. 0-500 TL ( )
	2. 500-1000 TL ( )
	3. 1000-1500 TL ( )
	4. 1500-2000 ( )
	5. 2000-….. ( )
9. Hastalık başlangıç yaşı	
10. Hastalık süresi:	………./ay
11. Tedavisiz geçen süre:	………./ay
12. En son kullandığı düzenli tedavinin süresi:	………./ay
13. Hastanede yatış sayısı	1.Yatışı yok( )
	2.Tek yatış( )
	3.İki yatış( )
	4.Üç yatış( )
	5.Dört ve üzeri yatış( )
14. Ailede psikiyatrik hastalık öyküsü	1.Yok ( )
	2.Var ( )
15. Ailede psikotik bozukluk öyküsü	1.Yok ( )

	2.Var ( )
16. Ailede duygudurum bozukluğu öyküsü	1.Yok ( )
	2.Var ( )
17. İntihar öyküsü	1.Yok ( )
	2.Var ( )
18. Kendine zarar verici davranış-self mutilizm	1.Yok ( )
	2.Var ( )
19. Ek hastalık-madde kullanımı öyküsü	0. alkol kullanımı<br>1. madde kullanımı<br>2. sigara kullanımı<br>3. gebelik<br>4. hepatit<br>5. hiv<br>6. diğer………
20.Şu an tedavi alıyor mu?	1. Evet ( )<br>2. Hayır ( )
21.Şu an klinik	1. Alevli ( )<br>2. Kısmi remisyon ( )<br>3. Tam remisyon ( )
22.PANSS skoru	1. Pozitif Ölçek:<br>2. Negatif Ölçek:<br>3. Genel Psikopatoloji Ölçeği:<br>4.Toplam Puan:
23.Serum BDNF Düzeyi:	
